# A sucrose non‐fermenting‐1‐related protein kinase‐1 gene, *IbSnRK1*, improves starch content, composition, granule size, degree of crystallinity and gelatinization in transgenic sweet potato

**DOI:** 10.1111/pbi.12944

**Published:** 2018-05-29

**Authors:** Zhitong Ren, Shaozhen He, Ning Zhao, Hong Zhai, Qingchang Liu

**Affiliations:** ^1^ Key Laboratory of Sweetpotato Biology and Biotechnology Ministry of Agriculture/Beijing Key Laboratory of Crop Genetic Improvement/Laboratory of Crop Heterosis and Utilization Ministry of Education College of Agronomy & Biotechnology China Agricultural University Beijing China; ^2^ College of Agronomy Qingdao Agricultural University Qingdao China

**Keywords:** *IbSnRK1*, starch content, starch quality, sweet potato

## Abstract

Sucrose non‐fermenting‐1‐related protein kinase‐1 (SnRK1) is an essential energy‐sensing regulator and plays a key role in the global control of carbohydrate metabolism. The *SnRK1* gene has been found to increase starch accumulation in several plant species. However, its roles in improving starch quality have not been reported to date. In this study, we found that the *IbSnRK1* gene was highly expressed in the storage roots of sweet potato and strongly induced by exogenous sucrose. Its expression followed the circandian rhythm. Its overexpression not only increased starch content, but also decreased proportion of amylose, enlarged granule size and improved degree of crystallinity and gelatinization in transgenic sweet potato, which revealed, for the first time, the important roles of *SnRK1* in improving starch quality of plants. The genes involved in starch biosynthesis pathway were systematically up‐regulated, and the content of ADP‐glucose as an important precursor for starch biosynthesis and the activities of key enzymes were significantly increased in transgenic sweet potato. These findings indicate that *IbSnRK1* improves starch content and quality through systematical up‐regulation of the genes and the increase in key enzyme activities involved in starch biosynthesis pathway in transgenic sweet potato. This gene has the potential to improve starch content and quality in sweet potato and other plants.

## Introduction

In plants, starch is the most important storage carbohydrate, which is widely used in our life and industries (Horrer *et al*., 2016; Thalmann *et al*., 2016; Zeeman *et al*., 2010). Starch usually exists in crop plants storage tissues such as endosperms and storage tubers/roots. Its production is critical to both yield and quality of crops, and high content and quality of starch in crops have become the main targets of breeders (Burrell, 2003; Volpicella *et al*., 2016).

Starch in plants has two forms of glucan polymer, amylose (10%–25%) and amylopectin (75%–90%) (Tester *et al*., 2004). Amylose is a linear or infrequently branched molecule which is composed of α‐1, 4‐linked D‐glucosyl units and <1% α‐1,‐6 linkages. Amylopectin is a highly branched glucan which consists of short α‐1,‐4‐linked D‐glucosyl chains with 5%–6% α‐1, 6 linkages (Ball and Morell, 2003; James *et al*., 2003; Tetlow *et al*., 2004). Starch biosynthesis is a complicated process, and a series of enzymes are generally considered to be linked with its biosynthesis, including invertase (acid invertase and neutral invertase), sucrose synthase (SuSy), ADP‐glucose pyrophosphorylase (AGPase), granule‐bound starch synthase (GBSS, also called Waxy), soluble starch synthase (SS), starch‐branching enzyme (SBE) and starch‐debranching enzyme (DBE) (Abe *et al*., 2013; James *et al*., 2003).

Sucrose non‐fermenting‐1‐related protein kinase‐1 (SnRK1) is an essential energy‐sensing regulator and plays a key role in the global control of carbohydrate metabolism (Halford and Hrdie, 1998). The genes encoding SnRK1 have been cloned from several plant species such as rye (Alderson *et al*., 1991), potato (Lakatos *et al*., 1999), *Arabidopsis* (Kleinow *et al*., 2000), maize (Lumbreras *et al*., 2001), sorghum (Jain *et al*., 2008) and *Malus hupehensis* Rehd. (Li *et al*., 2010). Antisense inhibition of *SnRK1* in developing pollen grains caused an almost complete loss of starch accumulation in transgenic barley (Zhang *et al*., 2001). Kanegae *et al*. (2005) found that *SnRK1* had a role in starch accumulation of rice. Starch level was increased by 30% in the tubers of the *SnRK1*‐overexpressing potato plants (McKibbin *et al*., 2006). The soluble sugar and starch contents were significantly increased in transgenic tomato overexpressing a heterologous *SnRK1* gene from *M. hupehensis* Rehd. (Wang *et al*., 2012). However, roles of *SnRK1* in improving starch quality have not been reported to date.

Sweet potato, *Ipomoea batatas* (L.) Lam., is an important starch crop. Its potential as food and carbohydrate source has been widely recognized (Jarret *et al*., 1992; Jiang *et al*., 2013). Improving starch content and quality of this crop remains an urgent demand, especially in the field of biotechnology. Several genes encoding the starch biosynthesis enzymes, including *GBSSI*,* SSI*,* SBEI* and *SBEII*, have been isolated from sweet potato and their overexpression or down‐regulation by RNAi impacts starch content, composition and physicochemical properties of this crop (Hamada *et al*., 2006; Kimura *et al*., 2001; Otani *et al*., 2007; Shimada *et al*., 2006; Wang *et al*., 2017b; Zhou *et al*., 2015). Tanaka *et al*. (2009) found that the storage root dry matter content and starch content were significantly increased in transgenic sweet potato overexpressing *SRF1* encoding a Dof zinc finger transcription factor. Overexpression of *IbAATP* encoding a plastidic ATP/ADP transporter protein significantly increased starch and amylose contents, enlarged starch granules and altered fine structure of amylopectin in transgenic sweet potato (Wang *et al*., 2016b).

In our previous study, one *IbSnRK1* gene was isolated from sweet potato cv. Lushu 3 (Jiang *et al*., 2013), and its overexpression increased the nitrate N content in roots and soluble protein and starch contents in leaves of transgenic sweet potato (Ren *et al*., 2018). In this study, we found that its overexpression not only increased starch content, but also decreased proportion of amylose, enlarged granule size and improved degree of crystallinity and gelatinization in the storage roots of transgenic sweet potato.

## Results

### Expression of *IbSnRK1* in sweet potato

**Figure 1:**
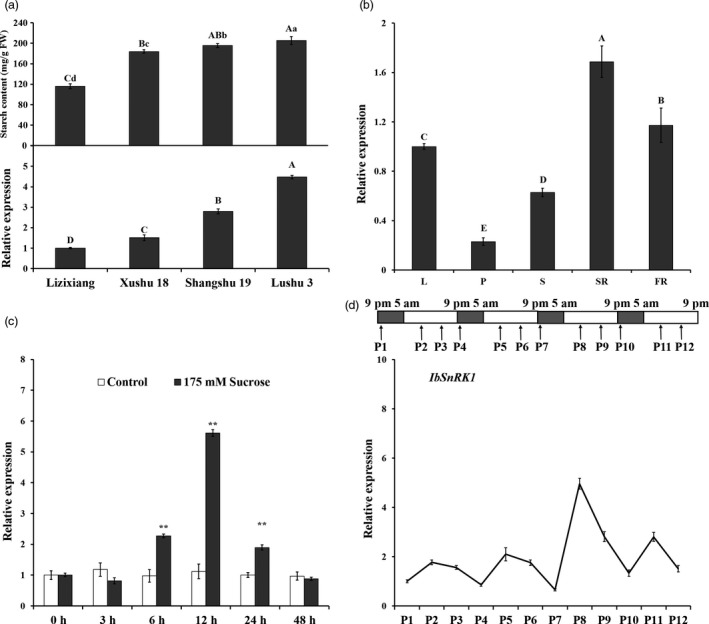
Expression analysis of IbSnRK1 in sweet potato. (a) Starch content and IbSnRK1 expression in the storage roots of different cultivars. (b) Expression of IbSnRK1 in different tissues of Lushu 3. L, leaf; P, petiole; S, stem; SR, storage root; FR, fibrous root. (c) Expression of IbSnRK1 in response to 175 mm sucrose treatment. The treatment with H_2_O was used as control. (d) Time settings used to examine the circadian rhythm during 16‐h light/8‐h dark photoperiods with light on at 5 am and off at 9 pm. Total RNA was extracted from the *in vitro* grown plants sampled at 10 pm (P1, P4, P7 and P10), 10 am (P2, P5, P8 and P11) and 4 pm (P3, P6, P9 and P12), respectively. The results are expressed as relative values with respect to P1 (set to 1.0). Data are presented as the mean ± SD (*n* = 3). * and different lowercase letters indicate a significant difference at *P* < 0.05; ** and different capital letters indicate a significant difference at *P* < 0.01 by Student's *t*‐test.

Real‐time quantitative PCR (qRT‐PCR) analysis showed that the expression levels of *IbSnRK1* in sweet potato cultivars Lizixiang, Xushu 18, Shangshu 19 and Lushu 3 with different starch content were positively correlated with their starch contents (Figure 1a). The highest expression level of *IbSnRK1* was found in the storage roots among various tissues of Lushu 3 (Figure 1b). The expression of *IbSnRK1* was strongly induced by exogenous sucrose and reached the highest level at 12 h after 175 mm sucrose treatment in the leaves of Lushu 3 (Figure 1c). Its expression in Lushu 3 followed the circandian rhythm and peaked at 10 am (P2, P5, P8 and P11) in each cycle, which displayed a sine‐wave pattern with the circadian rhythm treatment (Figure 1d).

### Production of the *IbSnRK1*‐overexpressing sweet potato plants

A total of 285 putatively transgenic plants were produced from the 1800 cell aggregates of sweet potato cv. Lizixing cocultivated with *Agrobacterium tumefaciens* (Figure S1a–e). GUS assay showed that 18 of them, named L1, L2, L3, …, L18, respectively, had visible GUS activity in leaf, stem and root tissues (Figure S1f–h). PCR analysis of genomic DNA confirmed the presence of *IbSnRK1* in the 18 GUS‐positive plants and the absence of *IbSnRK1* in the wild type (WT) and empty vector control (VC) (Figure S1i). The transgenic plants, WT and VC, were transferred to soils in a greenhouse and then in a field, and no morphological variations were observed among them (Figure S1j–m).

### Starch and amylose contents

**Figure 2:**
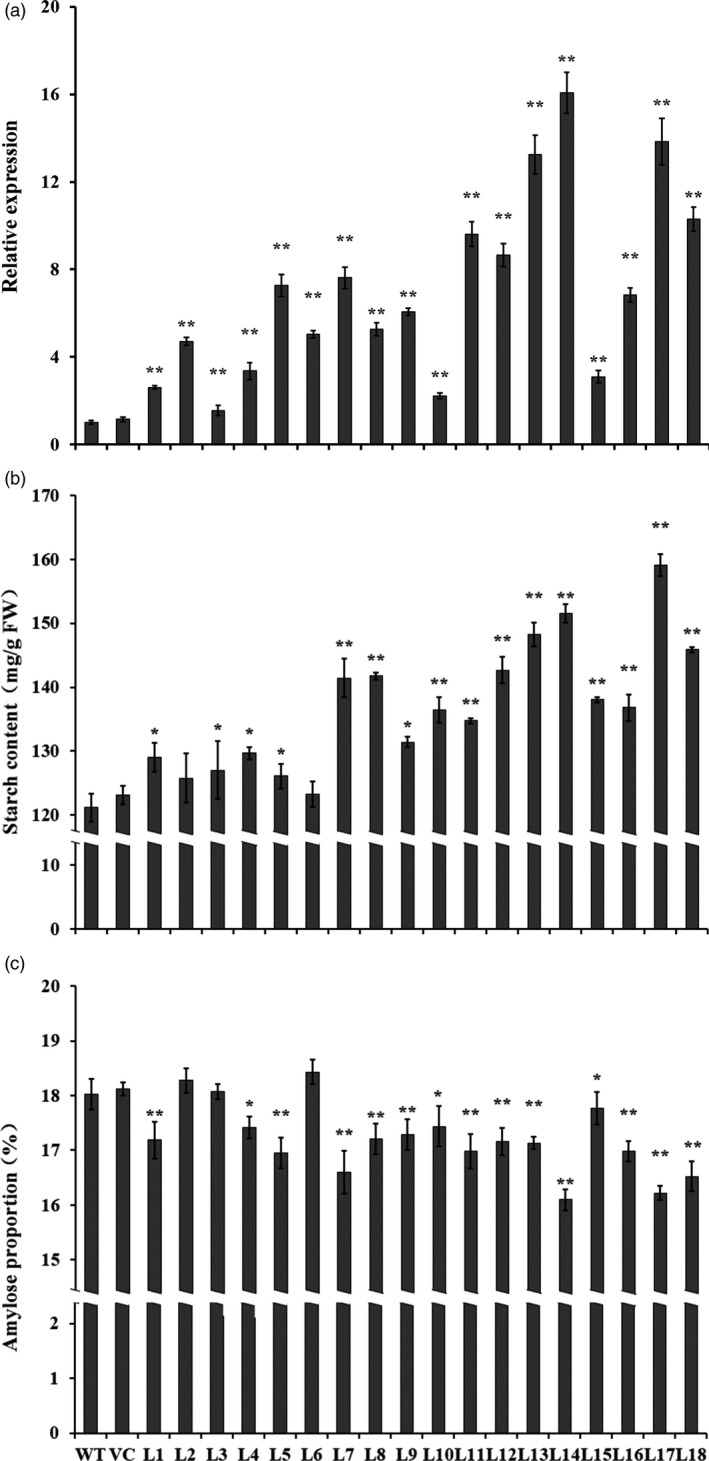
Expression analysis of IbSnRK1 (a), starch content (b) and amylose proportion (c) in the storage roots of the transgenic sweet potato plants, WT and VC. The sweet potato β‐actin gene was used as an internal control. Data are presented as the mean ± SD (*n* = 3). * and ** indicate a significant difference compared with WT at *P* < 0.05 and *P* < 0.01, respectively, by Student's *t*‐test.

qRT‐PCR analysis showed that the expression levels of *IbSnRK1* were significantly higher in the transgenic sweet potato plants than in WT and VC (Figure 2a). The starch content in the transgenic plant storage roots was increased by 1.7%–31.3% compared with WT, while the proportion of amylose (except for L2, L3 and L6) was decreased to 16.1%–17.8% from 18.0% of WT (decreased by 1.4%–10.7%) (Figure 2b,c). No differences in starch and amylose contents were found between WT and VC (Figure 2b,c).

### Starch granule morphology and size

**Figure 3:**
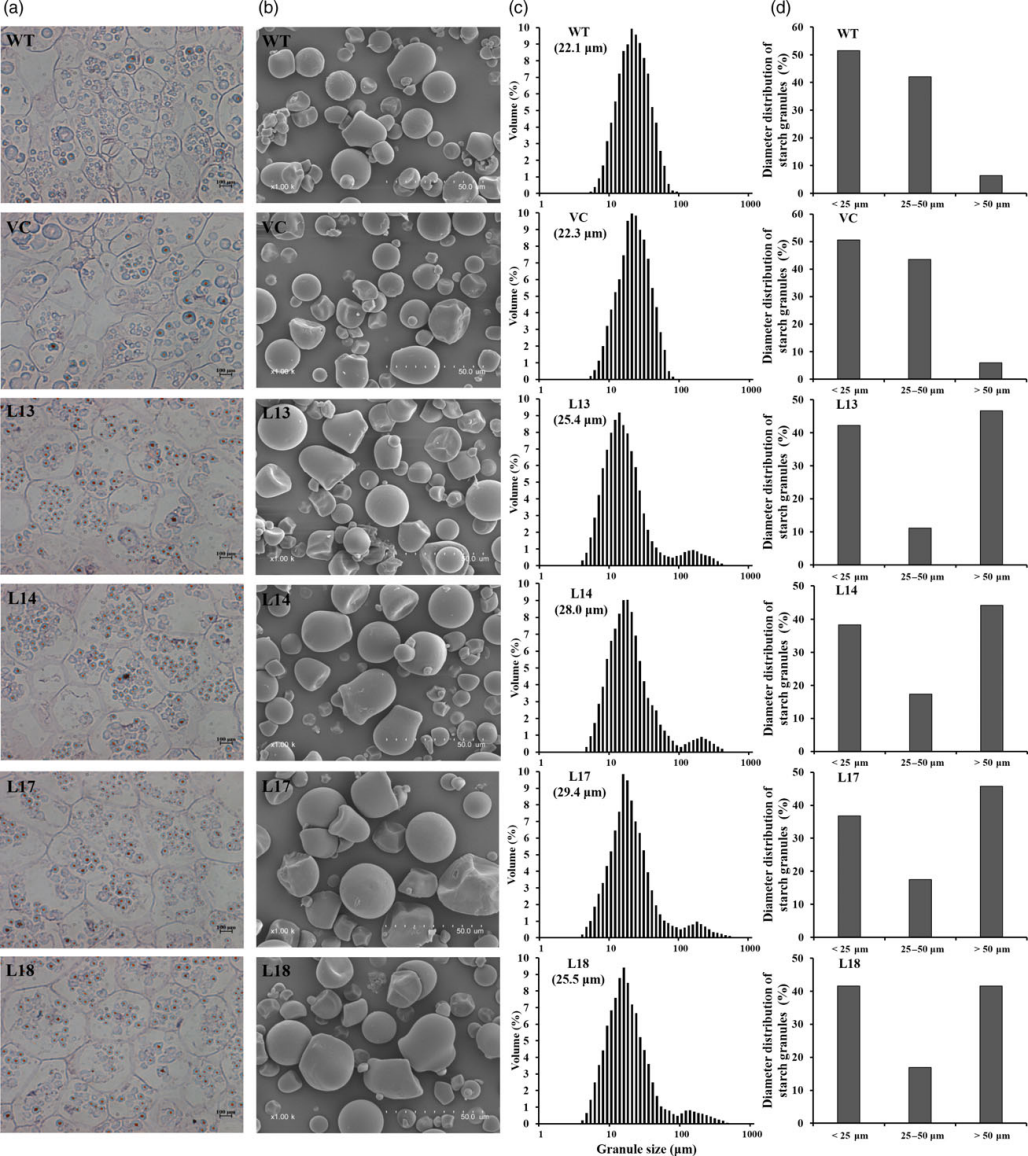
Morphology and size distribution of starch granules from the storage roots of the transgenic sweet potato plants, WT and VC. (a) Starch granules in amyloplasts. The bar indicates a length of 100 μm. (b) Scanning electron micrographs of the starch granules. The dotted line indicates a length of 50 μm. (c) Size distribution and mean volume diameter (MV) of starch granules. (d) Comparison of the starch granule diameter.

Four transgenic sweet potato plants with the highest starch content, L13, L14, L17 and L18, were selected for their starch quality evaluation. The number of starch granules in the amyloplasts of freshly harvested storage roots of the four transgenic plants was obviously increased compared with WT and VC (Figure 3a), but no variations in morphology of starch granules were observed among them (Figure 3b). Starch granule diameters of WT and VC were 5.2–76.0 μm and 5.2–66.9 μm, with a mean volume diameter (MV) of 22.1 and 22.3 μm, respectively, and their distribution displayed a unimodal pattern with a single peak at approximately 18 μm (Figure 3c). Most of the starch granules in WT and VC were <50 μm in diameter, and the proportions of <25 μm, 25–50 μm and >50 μm in WT were 51.5%, 42.1% and 6.4%, respectively, and in VC 50.5%, 43.5% and 6.0%, respectively (Figure 3d). In the transgenic plants, starch granule sizes ranged from 4.0 to 400.0 μm, with the MV of 25.4 to 29.4 μm, and their distributions exhibited two peaks at approximately 16 and 130 μm (Figure 3c). There were no differences in the proportion of <25 μm between the transgenic plants and WT/VC, but the proportions of 25–50 μm were decreased to 11.2%–17.5% and the proportions of >50 μm were increased to 41.6%–46.6% (Figure 3d).

### Chain length distribution

**Figure 4:**
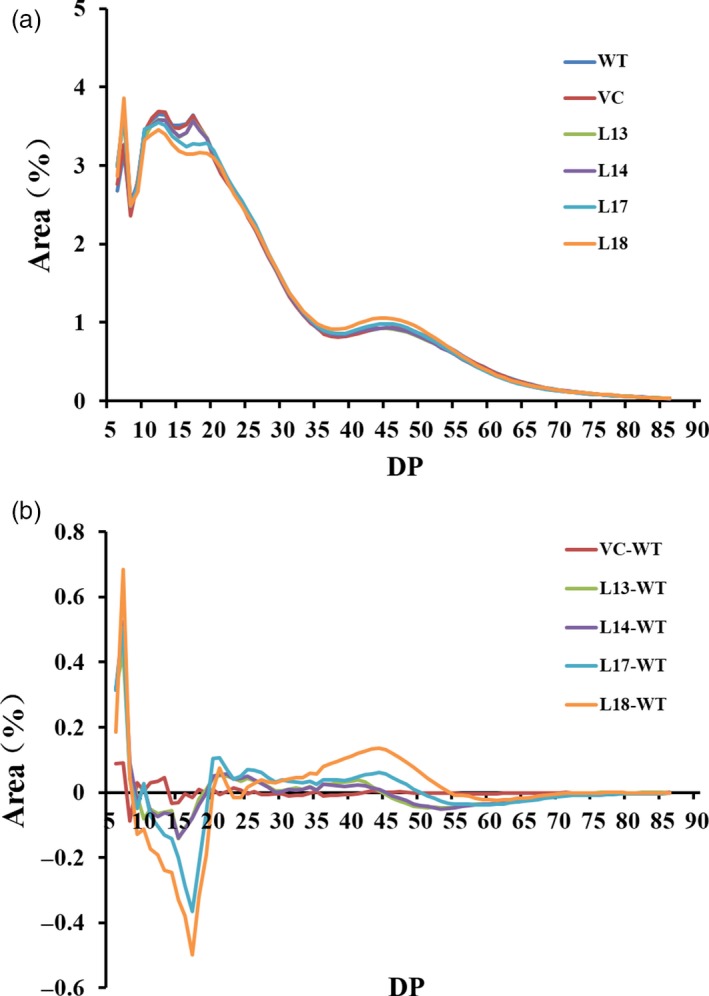
Chain length distribution (CLD) of amylopectin from the transgenic sweet potato plants, WT and VC. (a) CLD of the amylopectin after normalization to the total peak area. (b) Differences of CLD between the transgenic plants and WT were calculated as follow: the normalized CLD value for each transgenic plant and VC minus the value obtained for WT. DP, degree of polymerization.

Chain length distribution (CLD) of amylopectin exhibited a similar pattern in the four transgenic plants and WT/VC, and four peaks were present at approximately degree of polymerization (DP) 7, DP 12, DP 17 and DP 46 in L13, L14, WT and VC, but only three peaks (DP 7, DP 12 and DP 46) were found in L17 and L18 (Figure 4a). The value for the amylopectin glucan chain of WT was subtracted from the corresponding value of the transgenic plants to generate the CLD difference models (Figure 4b). The results indicated that the number of chains with DP 5–8 was markedly increased, but that of DP 9–19 was decreased, followed by the increase in DP 20–48 (Figure 4b).

### Analysis of X‐ray diffraction

**Figure 5:**
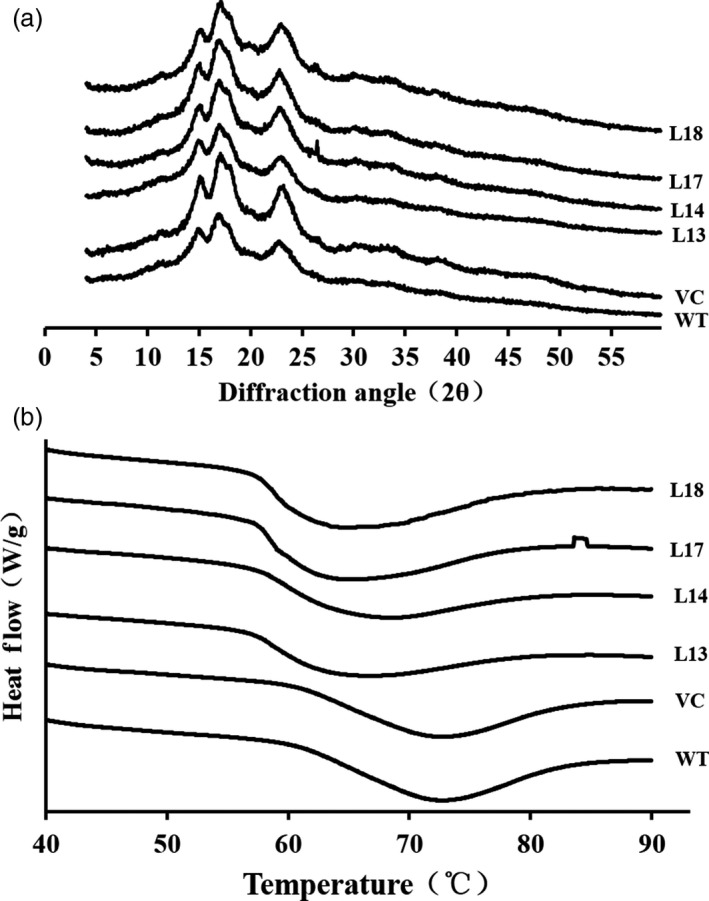
Wide‐angle X‐ray powder diffraction spectra (a) and differential scanning calorimeter thermograms (b) of starches from the storage roots of the transgenic sweet potato plants, WT and VC.

X‐ray diffractograms showed that starch from the transgenic sweet potato plants, WT and VC, exhibited a similar pattern with three strong reflections at 2θ of about 15°, 17° and 23° (Figure 5a), which indicated their crystal types were all A‐type (Table S1). Degree of crystallinity of starch from WT and VC was 42.1% and 41.8%, respectively, but that of the transgenic plants was increased to 44.9%–47.4% (Table S1).

### Thermal characterization

The transgenic sweet potato plants exhibited different patterns of starch gelatinization from WT and VC (Figure 5b). The starch of WT gelatinized at a temperature range of 60.2 °C (onset tempertature, *T*_o_) to 82.4 °C (conclusion temperature, *T*_c_) with an enthalpy (Δ*H*) of 11.1 J/g. However, *T*_o_, *T*_c_ and Δ*H* of starch from the transgenic plants were significantly decreased, indicating their starch was earlier to gelatinize, compared with WT (Table S2).

### Expression of starch biosynthesis genes

**Figure 6:**
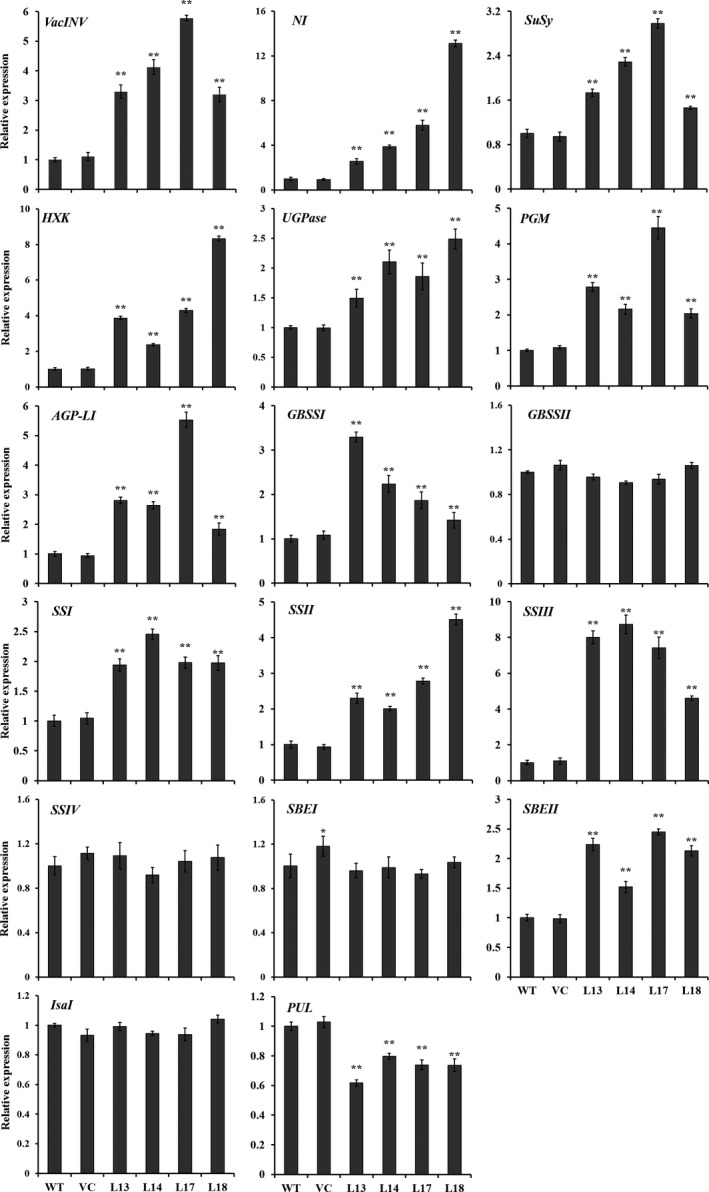
Expression of the genes involved in starch biosynthesis pathway in the storage roots of the transgenic sweet potato plants, WT and VC. Data are presented as the mean ± SD (*n* = 3). * and ** indicate a significant difference compared with WT at *P* < 0.05 and *P* < 0.01, respectively, by Student's *t*‐test.

The genes involved in starch biosynthesis pathway, including *VacINV*,* NI*,* SuSy*,* HXK*,* UGPase*,* PGM*,* AGP‐L1*,* GBSSI*,* SSI*,* SSII*,* SSIII* and *SBEII* (except for *GBSSII*,* SSIV* and *SBEI*) encoding acid invertase, neutral invertase, sucrose synthase, hexokinase, UDP‐glucose pyrophosphorylase, phosphoglucomutase, AGPase, GBSSI, SSI, SSII, SSIII and SBEII, respectively, were systematically up‐regulated in the storage roots of transgenic sweet potato plants compared with WT and VC (Figure 6). The transcript level of *PUL* encoding starch‐debranching pullulanase was significantly decreased, but *IsaI* encoding starch‐debranching isoamylase showed no change in expression level (Figure 6).

### The activities of starch biosynthesis key enzymes

**Figure 7:**
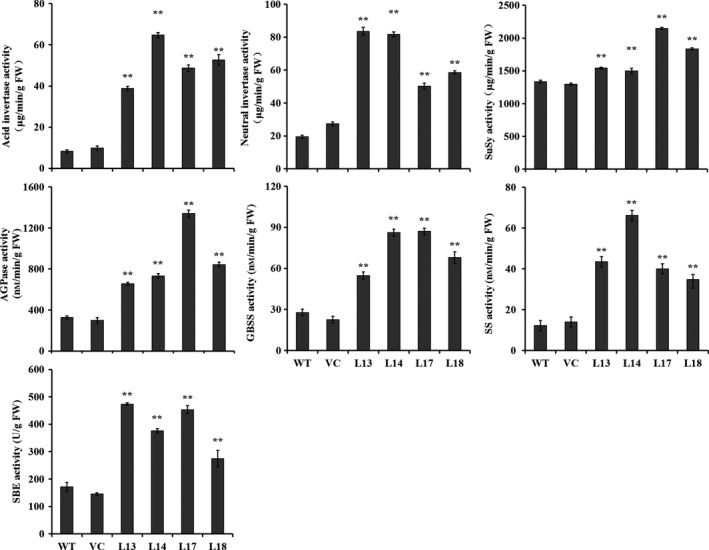
Enzyme activities of acid invertase, neutral invertase, SuSy, AGPase, GBSS, SS and SBE in the storage roots of the transgenic sweet potato plants, WT and VC. Data are presented as the mean ± SD (*n* = 3). * and ** indicate a significant difference compared with WT at *P* < 0.05 and *P* < 0.01, respectively, by Student's *t*‐test.

The activities of key enzymes, acid invertase, neutral invertase, SuSy, AGPase, SS, GBSS and SBE for starch biosynthesis were significantly increased to 7.3–12.2, 2.58–4.26, 1.12–1.61, 2.0–4.1, 2.0–3.1, 2.9–5.4 and 1.6–2.8 folds in the storage roots of the transgenic sweet potato plants compared with WT, which were consistent with the expression of the corresponding genes (Figures 6 and 7). No differences were found in the activities between WT and VC.

### The contents of components related to starch biosynthesis

**Figure 8:**
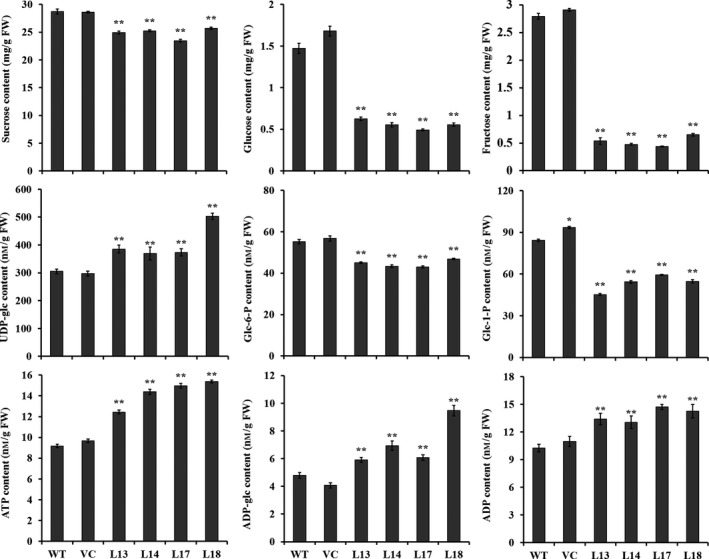
The content of components related to starch biosynthesis in the storage roots of the transgenic sweet potato plants, WT and VC. Data are presented as the mean ± SD (*n* = 3). * and ** indicate a significant difference compared with WT at *P* < 0.05 and *P* < 0.01, respectively, by Student's *t*‐test.

The contents of sucrose, glucose, fructose, glucose 6‐phosphate (glc‐6‐P) and glucose‐1‐phosphate (Glc‐1‐P) were significantly decreased, but those of uridine diphosphate glucose (UDP‐glc), adenosine triphosphate (ATP), adenosine diphosphate glucose (ADP‐glc) and ADP were significantly increased in transgenic sweet potato plants compared with WT (Figure 8). No differences in the content of these components were found between WT and VC (Figure 8).

## Discussion

The *SnRK1* gene has been shown to increase starch accumulation in several plant species such as barley (Zhang *et al*., 2001), rice (Kanegae *et al*., 2005) and potato (McKibbin *et al*., 2006), but its roles in improving starch quality of plants have not been reported to date. In the present study, the expression of *IbSnRK1* was strongly induced by exogenous sucrose (Figure 1c) and followed the circandian rhythm (Figure 1d). Endogenous circadian clock has been shown to affect starch accumulation in several plant species (Wang *et al*., 2001). Its overexpression not only increased starch content, but also decreased proportion of amylose, increased number of starch granules, enlarged mean granule size and improved degree of crystallinity and gelatinization in transgenic sweet potato. These findings reveal, for the first time, the important roles of *SnRK1* in improving starch quality of plants (Figures 3, 4, 5; Tables S1 and S2).

The content and composition of starch directly impact the starch yield and quality of crops (Bahaji *et al*., 2014; Burrell, 2003; Volpicella *et al*., 2016). The amylose–amylopectin ratio is a key factor that affects starch properties and is also important for food and bio‐industry applications of starches (Zeeman *et al*., 2010; Zhou *et al*., 2015). Low‐amylose starch is very important for optimizing industrial applications and allowing consumers to select crop varieties for health benefits (BeMiller and Whistler, 2009; Chung *et al*., 2011). Sweet potato starch is normally composed of 20%–30% amylose and 70%–80% amylopectin (Zhou *et al*., 2015). Its starch granules normally range from 4 to 40 μm, with an average size of 19 μm (Hoover, 2001). In this study, the content of starch and the number and size of starch granules were significantly increased, while the proportion of amylose was significantly decreased in the transgenic sweet potato lines (Figures 2 and 3). The increased number and size of starch granules might contribute to more accumulation of starch in the transgenic lines. By practical experience, the enlarged starch granules are beneficial to the extraction and separation of starch from the storage roots of sweet potato and its industrial applications.

There is a negative correlation between degree of crystallinity and amylose content, but a positive correlation between gelatinization and amylose content in plants (Cheetham and Tao, 1998; Copeland *et al*., 2009; Zhou *et al*., 2015). Our study showed that the four transgenic lines exhibited altered starch chain lengths, increased degree of crystallinity and decreased gelatinization temperature and enthalpy (Figure 4 and 5; Tables S1 and S2). These results indicated that the alternation of starch chain lengths led to the low proportion of amylose, which resulted in the increased degree of crystallinity and decreased gelatinization temperature and enthalpy in the transgenic lines.

As is well known, the starch biosynthesis is a sophisticated and systematic process (Ohdan *et al*., 2005). Sucrose can switch on the expression of starch biosynthesis‐related genes to enhance starch accumulation (Ahn *et al*., 2010; Hattori *et al*., 1991). Invertase (acid invertase and neutral invertase) and SuSy are the key enzymes for the conversion of sucrose to starch in starch biosynthesis pathway and catalyse the conversion of sucrose to glucose and fructose and the cleavage of sucrose and UDP to UDP‐glucose and fructose, respectively (Keeling and Myers, 2010; Koch, 2004; Roitsch and González, 2004). Hajirezaei *et al*. (2000) found that both invertase and SuSy exhibited significantly higher activities at different developmental stages of potato tubers. AGPase plays a critical role in the first committed step of starch biosynthesis and converts Glc‐1‐P into ADP‐glc, and ADP‐glc is the precursor for the biosynthesis of amylose and amylopectin (Yang *et al*., 2017). GBSS is mainly involved in the elongation of glucan chains in the amylose production, and Waxy mutants almost completely lack amylose (Sano *et al*., 1985). In addition to its roles in amylose biosynthesis, GBSSI is also found to be responsible for the extension of long glucans within the amylopectin fraction (Delrue *et al*., 1992; Maddelein *et al*., 1994; Van de Wal *et al*., 1998).

A series of enzymes, such as SS, SBE and DBE (isoamylase, Isa and pullulanase, PUL), work together to catalyse the biosynthesis of amylopectin (Abe *et al*., 2013; Facon *et al*., 2013; McMaugh *et al*., 2014; Pei *et al*., 2015). It is reported that *SSI*, SS*II*,* SSIII*,* SBEI*,* SBEII*,* Isa* and *PUL* were closely linked to the amylopectin chains. *SSI* increased the proportion of chains with DP 5–8 and DP ≥ 18 and reduced the proportion of chains with DP 9–17 in transgenic sweet potato (Wang *et al*., 2016b). *SSII* mainly synthesized amylopectin chains with DP 8–12 and DP 13–25 in sweet potato (Takahata *et al*., 2010). The deficiency of *OsSSIIIa* in the rice *duI* mutant led to the decrease in the chains with DP > 30 (Ryoo *et al*., 2007). Overexpression of *SBEII* increased the proportion with DP 6–12, particularly with DP 6, and decreased the gelatinization temperature in potato (Brummell *et al*., 2015).

Purcell *et al*. (1998) reported that *SnRK1* was involved in the control of *SuSy* in potato. McKibbin *et al*. (2006) found that overexpression of *StSnRK1* decreased glucose level and increased starch content by enhancing the activities of SuSy and AGPase in transgenic potato. *SbSnRK1α* from the wild potato species *Solanum berthaultii* had significant effects on StvacINV1‐associated sucrose degradation in transgenic potato (Lin *et al*., 2015). The expression levels of *SuSy* and *AGPase* were increased and the activities of both enzymes were also enhanced in the *IbSnRK1*‐overexpressing tobacco plants (Jiang *et al*., 2013). Overexpression of *StSnRK1* from potato up‐regulated *SuSy*,* AGPase* and *SS III* and enhanced the activities of the corresponding enzymes in transgenic tobacco (Wang *et al*., 2017b).

**Figure 9:**
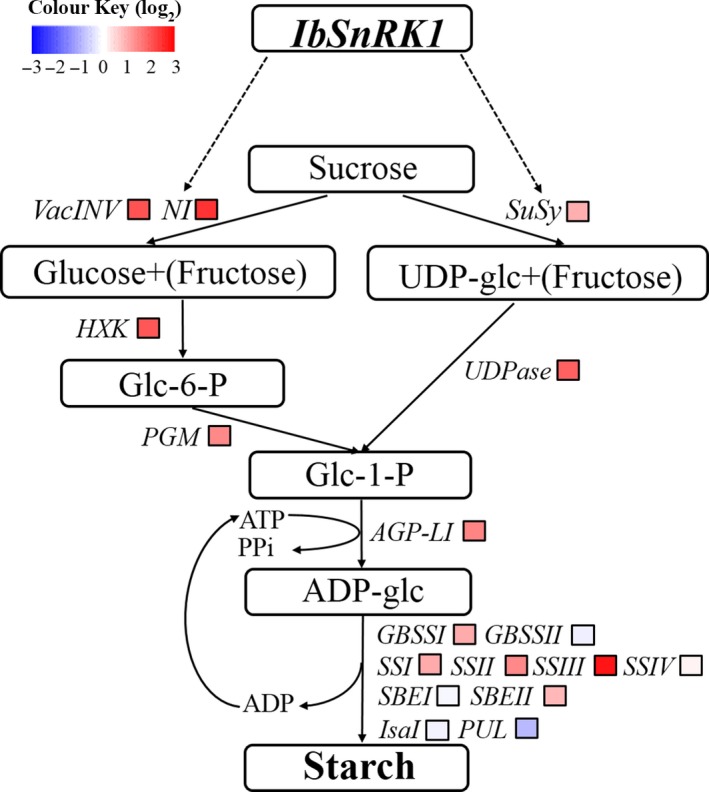
Diagram showing the regulation of starch biosynthesis in the storage roots of the IbSnRK1‐overexpressing sweet potato plants. Biosynthesis pathways are shown with solid arrows and regulatory interactions are shown with broken arrows. Fold changes (the mean of the four transgenic lines L13, L14, L17 and L18) are shown in colour, red boxes, white boxes and blue boxes indicate up‐regulation, no obvious change and down‐regulation of expression of genes encoding these enzymes (proteins), respectively.

In this study, most of the genes involved in starch biosynthesis pathway were systematically up‐regulated, the activities of key enzymes were increased, and the related components were also altered in the *IbSnRK1*‐overexpressing sweet potato plants (Figures 6, 7, 8). Our results support that *SnRK1* increases flux through the starch biosynthesis pathway by systematically up‐regulating the starch biosynthesis genes, which leads to increased starch content (Figure 9), as reported by Halford and Hey (2009). Furthermore, the significant up‐regulation of *SSI,* SS*II, SSIII* and *SBEII* responsible for amylopectin chains resulted in the increase in amylopectin proportion, which led to the increase in degree of crystallinity and the decrease in gelatinization temperature and enthalpy in the transgenic plants (Figure 5) (Brummell *et al*., 2015; Ryoo *et al*., 2007; Takahata *et al*., 2010; Wang *et al*., 2016b).

In conclusion, this study reveals, for the first time, the important roles of *SnRK1* in improving starch quality of plants. Its overexpression increased starch content, decreased proportion of amylose, enlarged granule size and improved degree of crystallinity and gelatinization by increasing the expression levels of genes and the activities of key enzymes involved in starch biosynthesis pathway in transgenic sweet potato. This gene has the potential to improve starch content and quality in sweet potato and other plants.

## Experimental procedures

### Plant materials

Sweet potato cultivars Lizixiang, Xushu 18, Shangshu 19 and Lushu 3 grown in a field were employed for analysing the expression of *SnRK1*. Lizixiang was used for characterizing the function of *IbSnRK1*.

### Expression analysis of *IbSnRK1* in sweet potato

Total RNA was isolated from the storage roots of four sweet potato cultivars and five tissues (leaf, petiole, stem, storage root and fibrous root) of Luhsu 3 using Trozol Kit (Transgen, Beijing, China). The first‐strand cDNA was synthesized using PrimeScript^™^ RT reagent Kit (TaKaRa, Dalian, China). qRT‐PCR was conducted to determine the transcript levels of *IbSnRK1* using SYBR Premix Ex Taq (TaKaRa, Dalian, China) with a 7500 Real‐Time PCR system (Applied Biosystems, Foster, CA). Specific primers of *IbSnRK1* and the sweet potato *Actin* (AY905538) gene as internal control were listed in Table S3. The gene expression was quantified using comparative *C*_T_ method (Schmittgen and Livak, 2008).

The response of *IbSnRK1* to exogenous sucrose was examined according to the method of Wang *et al*. (2016b). Leaf petioles from Lushu 3 plants grown in a field were treated for 1 day with water followed by treatments of either 175 mm sucrose or water (control) in the dark at 28 °C, and sampled at 0, 3, 6, 12, 24 and 48 h after treatment to analyse the expression of *IbSnRK1*.

The response of *IbSnRK1* to the circadian rhythm was investigated as described by Leterrier *et al*. (2008). *In vitro* grown plants of Lushu 3 were subjected to a 16‐h light/8‐h dark regimen for 1 month in a growth chamber at 28 °C prior to modifying the light conditions. Total RNA was extracted from each whole plant sampled at different time points (Figure 1d) for detecting the expression of *IbSnRK1* by qRT‐PCR.

### Production of the transgenic sweet potato plants

The expression vector pCAMBIA3301‐*IbSnRK1* contained *IbSnRK1* under the control of CaMV 35S promoter and NOS terminator and glucuronidase (*gusA*) and *bar* genes driven by a CaMV 35S promoter, respectively. Embryogenic suspension cultures of Lizixiang were prepared as described by Liu *et al*. (2001). Transgenic plants were produced using 0.5 mg/L phosphinothricin (PPT) as selection pressure according to the method of Yu *et al*. (2007) and identified by GUS assay and PCR analysis as described by Wang *et al*. (2016a). The transgenic plants, WT and VC, were transplanted in pots with a mixture of soil, vermiculite and humus (1:1:1, v/v/v) in a greenhouse and then grown in a field for further evaluation. The expression of *IbSnRK1* in the freshly harvested storage roots of the transgenic plants was analysed by qRT‐PCR as described above.

### Quantification of starch and amylose

The freshly harvested storage roots were employed to quantify starch content (Smith and Zeeman, 2006). Amylose content was determined using the colorimetric assay (Wang *et al*., 2016b). Standard curves were established with the standard samples of potato amylose and amylopectin (Sigma‐Aldrich, Shanghai, China).

### Analysis of starch granule morphology and size

Central pieces of the freshly harvested storage roots were used to prepare the paraffin sections (Li *et al*., 2017) and observed with a microscope (BX51 plus DP70, Olympus, Kyoto, Japan). The starch samples from the freshly harvested storage roots (Wang *et al*., 2017b) were spread on an aluminium stub using double‐sided adhesive tape, coated with gold and then observed with variable pressure scanning electron microscope (Hitachi S3400N, Tokyo, Japan) to examine the shape of starch granules. The starch granule size was measured with a Mastersize 2000 laser diffraction instrument (Malvern Instruments Ltd., Worcestershire, UK) according to the method of Zhou *et al*. (2015).

### Measurement of CLD

Starch samples were digested with isoamylase *Pseudomonas amyloderamosa* (Sigma‐Aldrich, Shanghai, China) as described by Nishi *et al*. (2001). CLD of amylopectin was analysed with high‐performance anion‐exchange chromatography with pulsed amperometric detection (HPAEC‐PAD, Dionex‐ICS 3000, Dionex Co., Sunnyvale, CA).

### Analysis of X‐ray diffraction

Starch samples were scanned through the 2θ range of 5–40° using a D8 Advance Bruker X‐ray diffractometer (Bruker AXS, Karlsruhe, Germany) to determine X‐ray diffraction. The crystal type and degree of crystallinity were calculated with Jade 5.0 software (Materials Data Inc., Livermore, CA).

### Analysis of thermal characteristics

Starch samples and distilled water were mixed (1:3, w/w), and the suspension was sealed in an aluminium pan with an empty aluminium pan as control for 24 h under room temperature. The samples were heated over a temperature range of 30–95 °C (raising the temperature at 10 °C/min) with a differential scanning calorimeter (DSC Q2000, TA Instruments Ltd., Crawley, UK), and the *T*_o_, peak temperature (*T*_p_), *T*_c_ and Δ*H* were measured with Universal Analysis 2000 (TA Instruments Ltd., Crawley, UK).

### Expression analysis of starch biosynthesis genes

Expression of starch biosynthesis‐related genes in the freshly harvested storage roots was analysed by qRT‐PCR as described above. The specific primers were listed in Table S3.

### Activity analysis of starch biosynthesis enzymes

The freshly harvested storage roots were grounded into power in liquid nitrogen. Activities of acid invertase, neutral invertase, SuSy, AGPase, GBSS, SS and SBE were measured according to the methods of Vargas *et al*. (2007), Baroja‐Fernández *et al*. (2012), Zhang *et al*. (2012) and Wang *et al*. (2016b), respectively.

### Quantification of components related to starch biosynthesis

The freshly harvested storage roots were used to quantify the contents of sucrose, glucose and fructose as described by Li *et al*. (2017). The contents of glc‐6‐P and glc‐1‐P were determined according to the method of Baroja‐Fernández *et al*. (2012). UDP‐glc, ADP‐glc, ATP and ADP were measured with the method of Gámez‐Arjona *et al*. (2011).

### Statistical analysis

All experiments were independently performed three times, and the data were presented as the mean ± SE. Results were analysed by Student's *t*‐test in a two‐tailed analysis using SPSS 20.0 Statistic Program. Significance was defined as *P *<* *0.05 (* or lowercase letters) and *P *<* *0.01 (** or capital letters), respectively.

## Conflict of interest

The authors declare no conflict of interest.

## Supporting information


**Figure S1** Production of transgenic sweet potato plants overexpressing the *IbSnRK1* gene.
**Table S1** X‐ray diffraction patterns of starches from the storage roots of the transgenic sweet potato plants, WT and VC.
**Table S2** The thermal characteristics of starches from the transgenic sweet potato plants, WT and VC.
**Table S3** Primers used in this study.
