# Supplementary material for: A sucrose non‐fermenting‐1‐related protein kinase‐1 gene, IbSnRK1, improves starch content, composition, granule size, degree of crystallinity and gelatinization in transgenic sweet potato
Source: Plant Biotechnol J. 2018 May 29;17(1):21–32. doi: 10.1111/pbi.12944 (PMC6330544; doi:10.1111/pbi.12944)
Supplement: Supplementary file 1 — Figure S1 Production of transgenic sweet potato plants overexpressing the IbSnRK1 gene. Table S1 X‐ray diffraction patterns of starches from the storage roots of the transgenic sweet potato plants, WT and VC. Table S2 The thermal characteristics of starches from the transgenic sweet potato plants, WT and VC. Table S3 Primers used in this study. [file PBI-17-21-s001.doc]

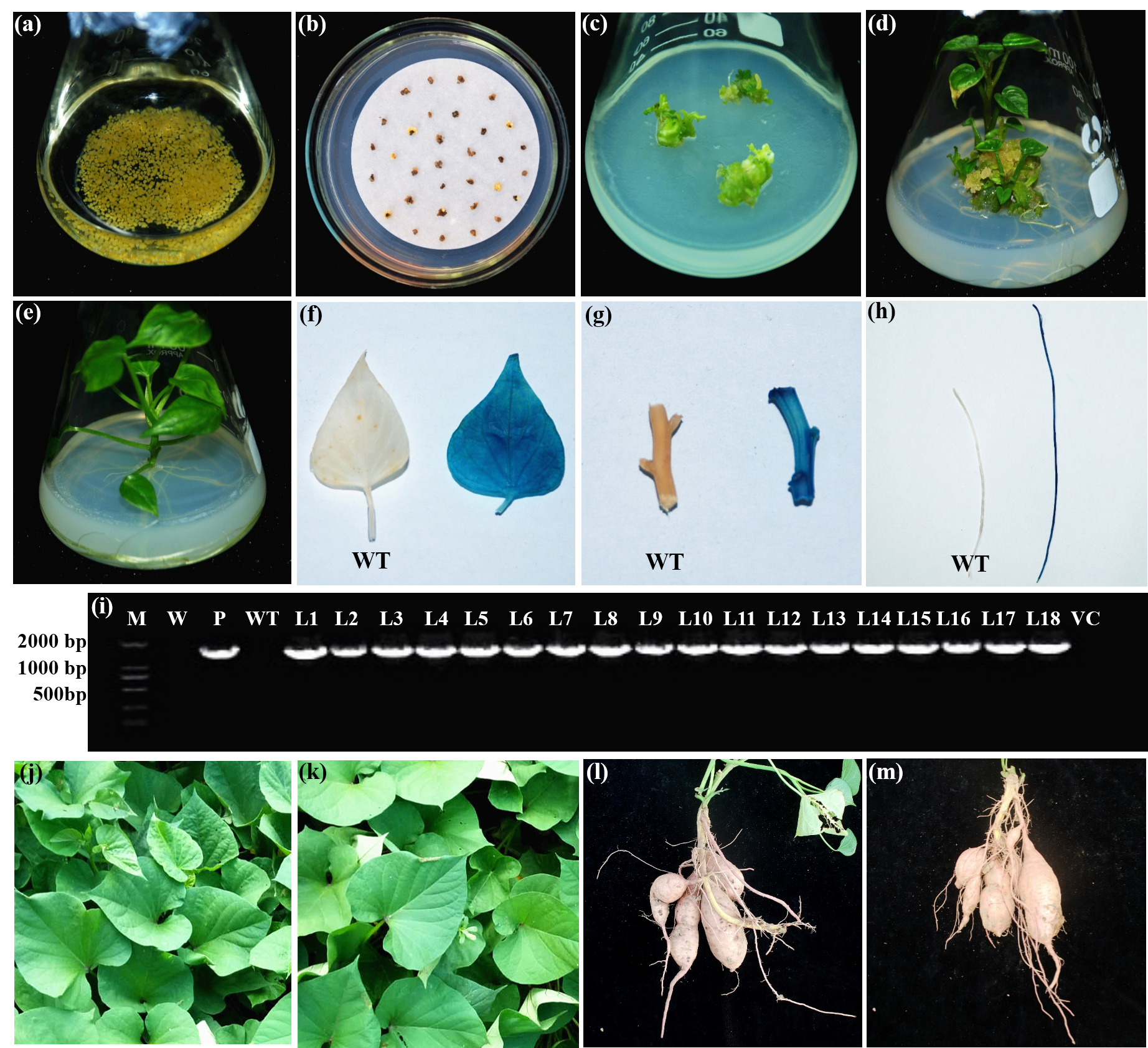


**Figure S1** Production of the *IbSnRK1*-overexpressing sweet potato plants. (a) Embryogenic suspension cultures rapidly proliferating in MS medium containing 2.0 mg /L 2,4-D. (b) PPT-resistant embryogenic calluses formed on MS medium with 2.0 mg /L 2,4-D, 100 mg /L Carb and 0.5 mg /L PPT after 8 weeks of selection. (c) and (d) Regeneration of plants from PPT-resistant calluses on MS medium with 1.0 mg /L ABA, 100 mg /L Carb and 0.5 mg /L PPT. (e) Whole plant formed on MS basal medium. (f), (g) and (h) GUS expression in leaf, stem and root of a transgenic plant and no GUS expression in WT, respectively. (i) PCR analysis of transgenic plants. M, DL2000 DNA marker; W, water as negative control; P, plasmid pCAMBIA3301-*IbSnRK1* as positive control; WT, negative control; L1-L18, transgenic plants; VC, empty vector control. (j) and (k) WT and transgenic plants grown in a greenhouse, respectively. (l) and (m) WT and transgenic plants grown in a field, respectively.

**Table S1 X-ray diffraction patterns of starches from the storage roots of the transgenic sweet potato plants, WT and VC**

| Lines | Diffraction peaks at 2*θ* value (°angle) | | | Degree of crystallinity (%) | Crystal pattern |
| --- | --- | --- | --- | --- | --- |
|  | 15 | 17 | 23 |  |  |
| WT | 70.5^a^(5.9)^b^ | 100(5.2) | 61.3(3.9) | 42.1^c^ | A  A  A  A  A  A |
| VC | 84.1(5.9) | 100(5.2) | 89.9(3.8) | 41.8 |  |
| L13 | 76.5(5.9) | 100(5.3) | 75.0(3.9) | 45.3 |  |
| L14 | 67.5(5.7) | 100(5.2) | 78.9(3.9) | 44.9 |  |
| L17 | 65.0(5.9) | 100(5.1) | 72.2(3.9) | 47.4 |  |
| L18 | 43.4(5.8) | 100(5.2) | 67.6(3.9) | 45.9 |  |

^a^ Relative intensity.

^b^ The values in parentheses represent interplanar spacings.

^c^ The apparent crystallinity estimated using Jade 5.0 software.

**Table S2 The thermal characteristics of starches from the transgenic sweet potato plants, WT and VC**

| Lines | *T_O_*（°C） | *T_P_*（°C） | *T_C_*（°C） | *ΔT*（°C） | *ΔH*（W /g） |
| --- | --- | --- | --- | --- | --- |
| WT | 60.2±0.1 | 72.0±0.1 | 82.4±0.4 | 22.2±0.2 | 11.1±0.6 |
| VC | 60.4±0.1 | 72.2±0.5 | 82.5±0.4 | 22.1±0.2 | 11.7±0.1 |
| L13 | 56.7±0.3^**^ | 64.0±0.3^**^ | 78.4±0.2^**^ | 21.7±0.4^*^ | 10.5±0.6^**^ |
| L14 | 56.9±0.2^**^ | 65.4±0.3^**^ | 78.5±0.4^**^ | 21.6±0.2^*^ | 9.5±0.6^**^ |
| L17 | 57.1±0.3^**^ | 63.5±0.3^**^ | 77.9±0.6^**^ | 20.8±0.8^**^ | 10.8±0.4^*^ |
| L18 | 57.1±0.1^**^ | 63.8±0.2^**^ | 77.9±0.5^**^ | 20.8±0.5^**^ | 10.7±0.4^*^ |

Data are presented as means±SD (n = 3). * and ** indicate a significant difference compared with WT at *P*<0.05 and *P*<0.01, respectively, by Student’s *t*-test.

**Table S3 Primers used in this study**

| Primer name | Primer sequence (5′-3′) |
| --- | --- |
| Primers for constructing expression vector |  |
| *IbSnRK1*-OE-F | CGGGATCCATGGATAGCAGAGGAGGTGG |
| *IbSnRK1*-OE-R | CGAGCTCCTAAGAGACTTTGAGATGGACAATA |
| Primers for identifying transformants |  |
| 35S-F | GAGGCTTACGCAGCAGGTC |
| *IbSnRK1*-R | CTAAGAGACTTTGAGATGGACAATA |
| Primers for qRT-PCR |  |
| *Actin*-F | AGCAGCATGAAGATTAAGGTTGTAGCAC |
| *Actin*-R | TGGAAAATTAGAAGCACTTCCTGTGAAC |
| *SnRK1-*F | TCTTAGTCCCAAGAGAAGAAAAAT |
| *SnRK1-*R | TAAATAAAATCTATTCAAGGCAATG |
| *VacINV-F* | GAAGAGGCAGTGGAATGTGAAGG |
| *VacINV-R* | AAGTAAACAGGGGTTAGCTCCGA |
| *NI-*F | ATGTGCCATTCTTGAGCGGA |
| *NI-*R | TTAAGCGATGCCGTAACCGA |
| *SuSy-*F | AGCAATCTGCAAAGAGGACCA |
| *SuSy-*R | TCTCACATATTCCCAAACACCAG |
| *HXK-*F | GCAAGTTGTCGACGCTATGG |
| *HXK-*R | AGGAATTGGCTGCTGCTGTA |
| *UGPase-*F | GAACAGTGGTAAACTTGATGCCCT |
| *UGPase-*R | CATCAGGGACTTGGGCTATTTC |
| *PGM-*F | TACCGTCGCCGAATCTCAAG |
| *PGM-*R | ATCACCTCCGAGAACCAACAACT |
| *AGP-LI*-F | GAGATATCCCACATCCAACGACTT |
| *AGP-LI*-R | TAGGGCCAAGTTAGCGTCGTAG |
| *GBSSI-*F | TGGCAACTATAACTGCCTCACAC |
| *GBSSI-*R | GGCACTGGTTCTCAATTGTAACAT |
| *GBSSII-*F | ACAAAGTCAGAGGATGGGTGGG |
| *GBSSII-*R | CAGCATGGACGACAGGGATAG |
| *SSI -*F | GCTGCAGACCGTCTTTGTGC |
| *SSI* -R | GAGCCATCCCTCTGTGCTCC |
| *SSII* -F | AGACTGTGGGATCTACTGAAAGGC |
| *SSII* -R | GTGAATCCACGTCCAGTGGC |
| *SSIII* -F | TCTGTTATCCTGAGGAGGTAAAACC |
| *SSIII* –R | CTCCCATGATCAATACATCAGGC |
| *SSIV -*F | CTGCTTTCTCATTTCTGTCATCGT |
| *SSIV -*R | GCTCAACTTCCACTTGACTCAGAG |
| *SBEI*-F | ATTCTTGGCCTAGACCAAGGG |
| *SBEI*-R | ACAATGCAGCCTTCTTCTTTGTTA |
| *SBEII*-F | AGTCCGCTGTTTGGAGGCTT |
| *SBEII*-R | CCTCAACTGGTTTTGCTTCGTC |
| *IsaI-F* | GGAACGAGGTGGTTATCGGTG |
| *IsaI-R* | TCTGGGCATAGCAACAGAATTATG |
| *PUL*-F | GCTGCTCGACGATGCCTCT |
| *PUL*-R | CATCCTCAACGTCCACATTCC |
